# Fertility factors affect the vaginal microbiome in women of reproductive age

**DOI:** 10.1111/aji.13220

**Published:** 2020-01-21

**Authors:** Jieying Xu, Gaorui Bian, Min Zheng, Gang Lu, Wai‐Yee Chan, Weiping Li, Kaiping Yang, Zi‐Jiang Chen, Yanzhi Du

**Affiliations:** ^1^ Center for Reproductive Medicine Ren Ji Hospital School of Medicine Shanghai Jiao Tong University Shanghai China; ^2^ Shanghai Key Laboratory for Assisted Reproduction and Reproductive Genetics Shanghai China; ^3^ Department of NGS Sequencing Tianyi Health Sciences Institute Zhenjiang China; ^4^ The Chinese University of Hong Kong‐Shandong University Joint Laboratory on Reproductive Genetics School of Biomedical Sciences The Chinese University of Hong Kong Hong Kong SAR China; ^5^ National Research Center for Assisted Reproductive Technology and Reproductive Genetics The Key Laboratory for Reproductive Endocrinology of Ministry of Education Shandong Provincial Key Laboratory of Reproductive Medicine Center for Reproductive Medicine Shandong Provincial Hospital Shandong University Jinan China; ^6^ Departments of Obstetrics & Gynaecology and Physiology & Pharmacology Children's Health Research Institute & Lawson Health Research Institute Western University London ON Canada

**Keywords:** fertility, microbiota, vagina

## Abstract

**Problem:**

For women of reproductive age, achieving a successful pregnancy requires both the normal functioning of reproductive endocrine and the health of the reproductive tract environment. We aimed to study how these fertility factors, such as female age, baseline sexual hormone levels, tubal patency, and vaginal pH, affect the composition of vaginal microbiome.

**Method of study:**

The 16S rRNA sequencing was carried on vaginal microbiome samples from 85 women of reproductive age without vaginal infections or reproductive endocrine diseases. The detailed correlations between fertility factors and vaginal microbiome were quantified by Spearman's rank tests. A linear discriminant analysis was carried out to explore the effects of fertility factors on the relative abundances of vaginal bacterial species.

**Results:**

The vaginal pH, levels of basal E2, LH, and FSH all had significant effects on the distribution of vaginal microbiome. The relative abundances of vaginal bacterial species, including *Escherichia coli*, *Streptococcus agalactiae*, and *Prevotella intermedia*, were significantly different due to the host's state of reproductive endocrine and tubal patency. It was worth noting that women with tubal obstruction, or prolonged menstrual cycle, or antral follicle count >15, or vaginal pH > 4.5 all had a higher abundance of *Escherichia coli* in vagina.

**Conclusion:**

The fertility factors associated with the reproductive endocrine and the genital tract environment affected vaginal microbiome in women of reproductive age. The species *Escherichia coli*, *Streptococcus agalactiae*, *Prevotella intermedia*, etc could be used as biomarkers to reflect the pathological state of reproductive endocrine and genital tract.

## Graphical abstract

The fertility factors associated with the reproductive endocrine and the genital tract environment affected vaginal microbiome in women of reproductive age, including the bacterial species *Escherichia coli*, *Streptococcus agalactiae*, and *Prevotella intermedia*, et al.

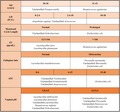

## 1 INTRODUCTION

For women of reproductive age, achieving a successful pregnancy requires both the normal functioning of reproductive endocrine and the health of the reproductive tract environment. Reproductive endocrine plays an essential role in promoting gonadal development, maintaining fertility, and ensuring reproductive health. Meanwhile, a healthy reproductive tract environment also helps support successful embryo implantation and maintains pregnancy.

As the age in women grows, the levels of sex hormones change and the vaginal microbiome is in constant flux.^1^ During a woman's reproductive years, the fluctuating levels of hormones that regulate the menstrual cycle are an important influence on the vaginal microbiome,^2^ especially the estradiol (E2) and progesterone (P).^3^, ^4^ The levels of sex hormones would affect the components of the female genital tract defensive barriers, including the mucous viscosity, epithelial barrier thickness, immune cell frequency, and resident vaginal microbes.^3^, ^5^

Previous studies have explored the physiological interaction between sex hormones and vaginal flora. Estrogen promotes hyperplasia and thickening of vaginal epithelia and the increase in glycogen.^6^ As the dominant bacteria in the vagina, *Lactobacillus* can change the glycogen into lactic acid, maintain the acidic environment of the vagina, inhibit the growth of other pathogens,^7^, ^8^ and strengthen the immune system.^9^, ^10^, ^11^ When a woman enters menopause, the level of estrogen will significantly reduce, while the level of follicle‐stimulating hormone (FSH) increases, and the vaginal pH environment changes from acidic to weakly acidic, which leads to the colonization of a large number of mixed bacteria.^12^, ^13^, ^14^ Meanwhile, the estradiol‐based hormone replacement therapy can maintain *Lactobacillus* dominance in post‐menopausal women, supporting a link between estradiol and lactobacilli.^12^, ^15^

The levels of sex hormones in the reproductive endocrine system are closely related to female fertility. Clinically, the levels of FSH, luteinizing hormone (LH), prolactin (PRL), testosterone (T), and antral follicle count (AFC) in the early follicular phase are used to assess the female fertility, as well as the female age, anti‐Müllerian hormone (AMH), and fallopian tube patency. However, there have been only a few studies about the correlation between these fertility factors and the vaginal microbiome.

Therefore, in this study, we examined the vaginal microbiome of women of reproductive age and tried to study how these fertility factors associated with the reproductive endocrine and reproductive tract environment affect the composition of the vaginal microbiome.

## 2 MATERIALS AND METHODS

### 2.1 Ethics statement

This study was approved by the Ethical Committee of Ren Ji Hospital, Shanghai Jiao Tong University School of Medicine (Approval No. 2016012904). All participants have provided written informed consent prior to their inclusion in the study.

### 2.2 Sample collection and clinical measurements

From December 2015 to April 2016, we recruited 85 women aged 24 to 43 years from the Center for Reproductive Medicine, Renji Hospital, Shanghai Jiao Tong University School of Medicine.

Among the 85 subjects recruited, 40 subjects were infertile due to the tubal obstruction. The other 45 subjects had normal fallopian tubes, who sought for the assisted reproductive technology support only due to the male sperm quality problems. Patients with reproductive endocrine diseases or had hormone replacement therapy in the past 3 months were excluded from this study.

Patients with bacterial vaginosis (Amsel criteria^16^), candidal vaginitis, trichomoniasis, mycoplasmal, chlamydial, or gonococcal infection were excluded. The subjects were given leukorrheal routine examinations. The pus cells of all subjects were 0‐15/HP, and the vaginal cleanliness was grade I–II. The *Candida albicans*, *Trichomonas vaginalis*, *Neisseria gonorrhoeae*, *Mycoplasma hominis*, *Ureaplasma urealyticum*, *Chlamydia trachomatis*, and clue cells were all not detected. The test results of hydrogen peroxide, leukocyte esterase, and sialic acid glucoside enzyme were all negative. Women who had taken any antibiotic drugs in the past 3 months were also excluded.

All participants reported their conditions of menstruation and fallopian tube patency. We recorded their age, height, and weight. The vaginal pH values were tested from the posterior fornix during the menstrual interval. We detected serum AMH levels using an ultrasensitive enzyme‐linked immunosorbent assay (Kangrun Biotech, catalog number KR‐AMH‐001). Since the levels of basic sexual hormones were regarded as stable at the baseline during the early follicular phase of the menstrual cycle,^17^ they were measured on day 1‐5 of the menstrual cycle by electrochemiluminescence immunoassay (ECLIA) (Roche Cobas E601), including FSH, LH, E2, PRL, and T. In the meantime, the antral follicle count (AFC), which means the sum of all follicles measuring 2‐10 mm in mean diameter in both ovaries, was done by a transvaginal ultrasound. The levels of clinical factors were displayed in histograms by SPSS software (version 22, SPSS Inc).

### 2.3 Total bacterial genomic DNA extraction

For each participant, the vaginal microbiome sample was obtained by a sterile cotton swab from the posterior fornix of the vagina during the menstrual interval. The vaginal bacterial DNA was extracted by a PowerSoil‐htp 96‐well soil DNA isolation kit (Mo Bio Laboratories, catalog number 12888‐100). DNA quantification was carried out with a NanoDrop spectrophotometer (Nyxor Biotech).

### 2.4 PCR amplification and 16S rRNA gene sequencing

Amplification and sequencing were carried out as protocols described by Mao et al^18^ The V4 regions of bacterial 16S rRNA genes were amplified using the primers 515F (5′‐barcode‐GTGCCAGCMGCCGCGGTAA‐3′) and 806R (5′‐barcode‐GGACTACHVGGGTWTCTAAT‐3′). Amplicons were purified using a Qiagen QIAquick PCR purification kit (Qiagen) and quantified using the PicoGreen dsDNA reagent kit (Invitrogen Ltd.). The pooled product was paired‐end sequenced (2 × 250) on an Illumina MiSeq platform by the Department of NGS Sequencing, Tianyi Health Sciences Institute Co., Ltd.. Sequence data were archived in the NCBI Sequence Read Archive (SRA) under accession number SRP136384.

### 2.5 Sequence processing and data analysis

Raw Illumina FASTQ files were demultiplexed, quality filtered, and analyzed by Quantitative Insights Into Microbial Ecology (QIIME, version 1.8.0).^19^ The operational taxonomic units (OTUs) were clustered with a 97% similarity cutoff using UPARSE.^20^ Taxonomy was assigned to representative sequences using the Ribosomal Database Project (RDP) classifier in QIIME^21^ with a confidence value of 0.8 against the Silva bacterial 16S rRNA gene dataset (version 119) and Greengenes database for species‐level taxonomy information.

### 2.6 Gene function prediction

We used Canoco 5.0 software to perform redundancy analysis (RDA) at the genus level.^22^ For the multiple comparisons, the false discovery rate (FDR) adjusting method was employed to avoid type I errors. Provided by Benjamini and Hochberg, it advocated control of the expected proportion of falsely rejected hypotheses.^23^ The correlation coefficients between the relative abundance of vaginal bacteria and clinical factors were calculated by Spearman's rank tests. Linear discriminant analysis (LDA) effect size (LEfSe) method was used to discover the high‐dimensional biomarker and explain the genomic feature identification that characterizes the differences between various biological conditions.^24^

## 3 RESULTS

### 3.1 Vaginal microbial community composition of the women of reproductive age

The clinical results of fertility factors of 85 women of reproductive age were shown in frequency bar charts (Figure S1). 16S rRNA gene sequencing generated a total of 3 218 048 high‐quality reads, which were binned into 18 796 OTUs after sequence alignment with the Greengenes database at 3% dissimilarity. The detailed sequence data were archived in the NCBI Sequence Read Archive (SRA) under accession number SRP136384.

**Figure 1:**
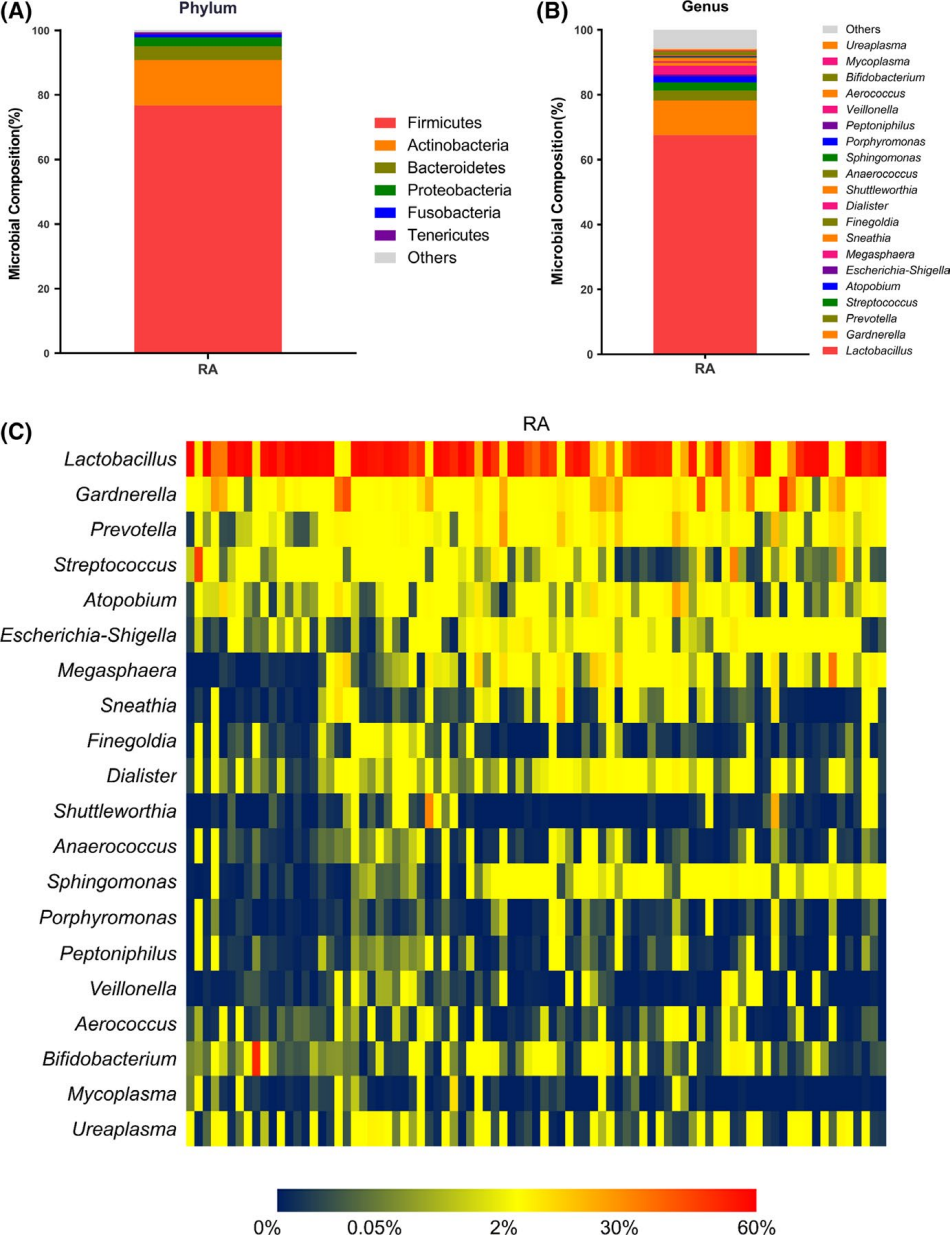
Results of 16S rRNA gene sequencing of the vaginal microbiome in women of reproductive age. The microbial composition of the vaginal microbiome at the phylum level (A) and the genus level (B). A heatmap was drawn from 20 dominant bacterial genera in all samples (C). RA, women of reproductive age

At the phylum level, Firmicutes were the principal strains in women of reproductive age, followed by Actinobacteria and Bacteroidetes (Figure 1A). At the genus level, the vaginal flora of women of reproductive age was dominated by *Lactobacillus*, followed by *Gardnerella*, *Prevotella*, *Streptococcus*, and *Atopobium* (Figure 1B). The heatmap showed the top 20 dominant genera in all samples (Figure 1C).

### 3.2 Analyses of correlations between the fertility factors and vaginal microbiome

**Figure 2:**
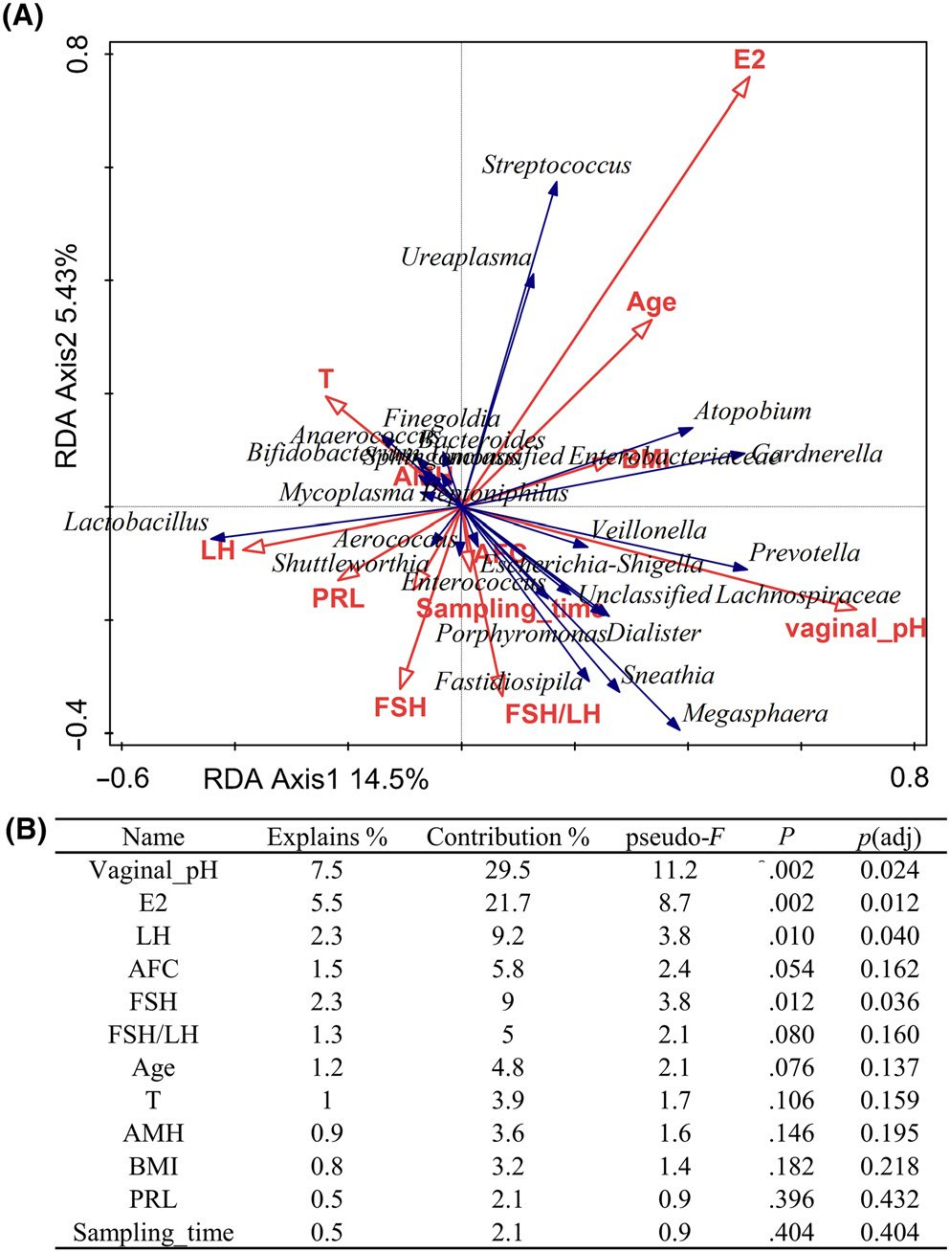
Redundancy analysis of the effects of fertility factors on the vaginal microbiome. A, A graphical result of redundancy analysis. Each point represented a single sample. The length of the arrow represented the correlation degree between the environmental factor and the sample distribution. An acute angle between the arrows indicated a positive correlation, while an obtuse angle indicated a negative correlation. B, The detailed contribution degree and the *P*‐value of each clinical factor. E2, estradiol. LH, luteinizing hormone. AFC, antral follicle count. FSH, follicle‐stimulating hormone. T, testosterone. AMH, anti‐Müllerian hormone. BMI, body mass index. PRL, prolactin

We used the redundancy analysis (RDA) to visually examine the effect of fertility factors on the distribution of samples (Figure 2). The results showed that vaginal pH, levels of serum E2, LH, and FSH all had significant effects on the distribution of vaginal microbiota (*P* < .05). Among them, vaginal pH and the levels of serum E2 had the most significant effects (contribution of 29.5% and 21.7%, respectively).

**Figure 3:**
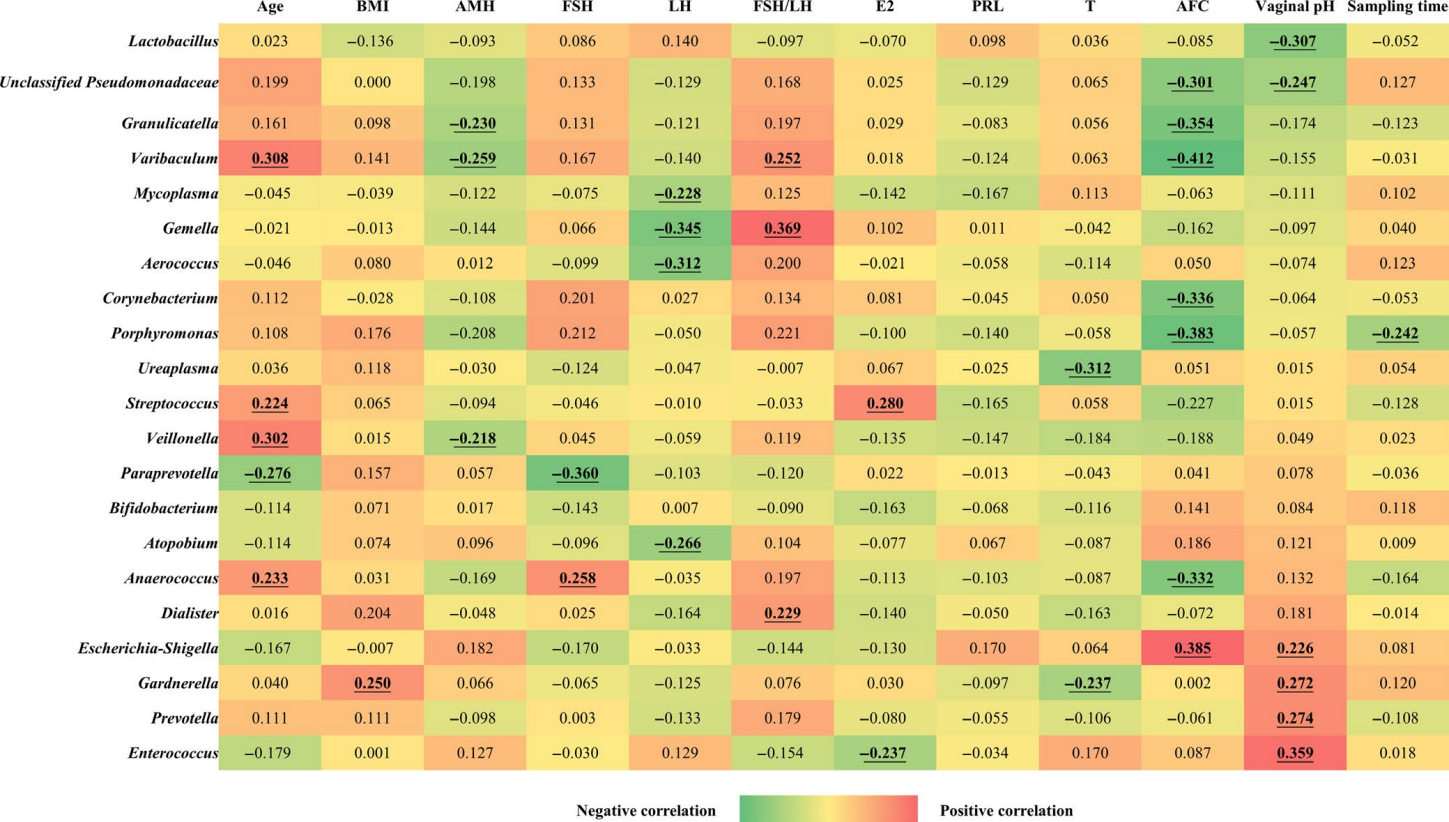
Spearman correlation analysis between the relative abundances of vaginal microbiome and clinical factors. The correlation coefficients were calculated between the relative abundances of vaginal bacterial genera and the clinical factors by Spearman's rank tests. The results with significant correlation (correlation coefficient > 0.2, *P* < .05) were presented in bold and underlined. Red indicated positive correlations while green indicated negative. BMI, body mass index. AMH, anti‐Müllerian hormone. FSH, follicle‐stimulating hormone. LH, luteinizing hormone. E2, estradiol. PRL, prolactin. T, testosterone. AFC, antral follicle count

Next, we quantified the correlations between the relative abundance of vaginal flora and fertility factors by Spearman's rank tests. The results were presented in a heatmap (Figure 3). The vaginal bacteria were arranged according to the value of the correlation coefficient between the relative abundance of vaginal bacteria and the vaginal pH.

As can be seen from the results, the relative abundance of *Lactobacillus* was negatively correlated with vaginal pH, suggesting that *Lactobacillus* was more likely to exist in a weakly acidic environment, which is a normal vaginal environment. Meanwhile, the relative abundances of *Escherichia/Shigella*, *Gardnerella*, *Prevotella*, and *Enterococcus* were positively correlated with vaginal pH. That means when the vaginal pH rises, the colonization of these bacteria would increase.

The relative abundance of *Paraprevotella* was negatively correlated with age and FSH. It was suggested that this bacterium was more commonly found in the vagina of women with normal ovarian function or younger. Meanwhile, the relative abundances of genera *Varibaculum*, *Streptococcus*, and *Veillonella* were positively related to age, indicating that the colonization of these bacteria in the vagina may increase with female age.

In addition, the relative abundances of genera *Aerococcus* and *Atopobium* were negatively correlated with LH. The relative abundance of *Gemella* was positively correlated with FSH/LH and that of *Streptococcus* was positively correlated with E2.

The vaginal genera negatively correlated with AFC included *Varibaculum*, *Porphyromonas*, *Granulicatella*, and *Anaerococcus*, while the relative abundance of *Escherichia/Shigella* was positively correlated with AFC.

### 3.3 Specific vaginal species in each subgroup of fertility factors

**Figure 4:**
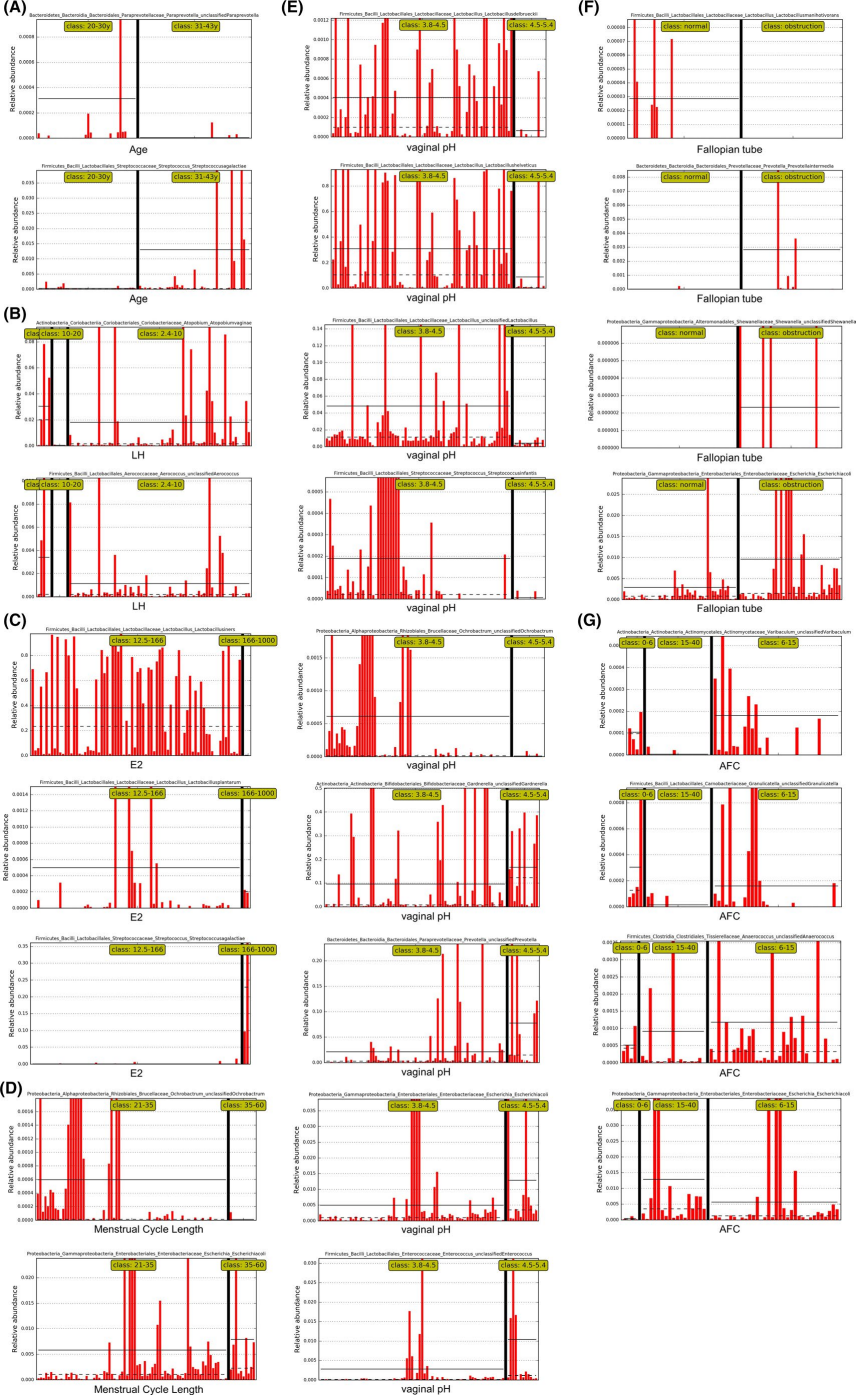
The relative abundances of specific vaginal species in each subgroup of fertility factors. The women of reproductive age were divided into multiple subgroups according to each fertility factor, including (A) age, (B) LH, (C) E2, (D) menstrual cycle length, (E) vaginal pH, (F) fallopian tube, and (G) AFC. The raw data of the features within each subgroup detected by LEfSe as biomarkers were plotted as abundance histograms (the logarithmic LDA score ≥ 2.0, *P* < .05). E2, estradiol. LH, luteinizing hormone. AFC, antral follicle count

We then divided the women of reproductive age into multiple subgroups based on each fertility factor. It was found that there were significant differences in the abundances of some vaginal species between/among the subgroups of several fertility factors, including the age, E2, LH, T, fallopian tube patency, menstrual cycle length, AFC, and vaginal pH. The results of linear discriminant analysis (LDA) are shown in Figure 4 (the logarithmic LDA score ≥ 2.0, *P* < .05).

**Figure 5:**
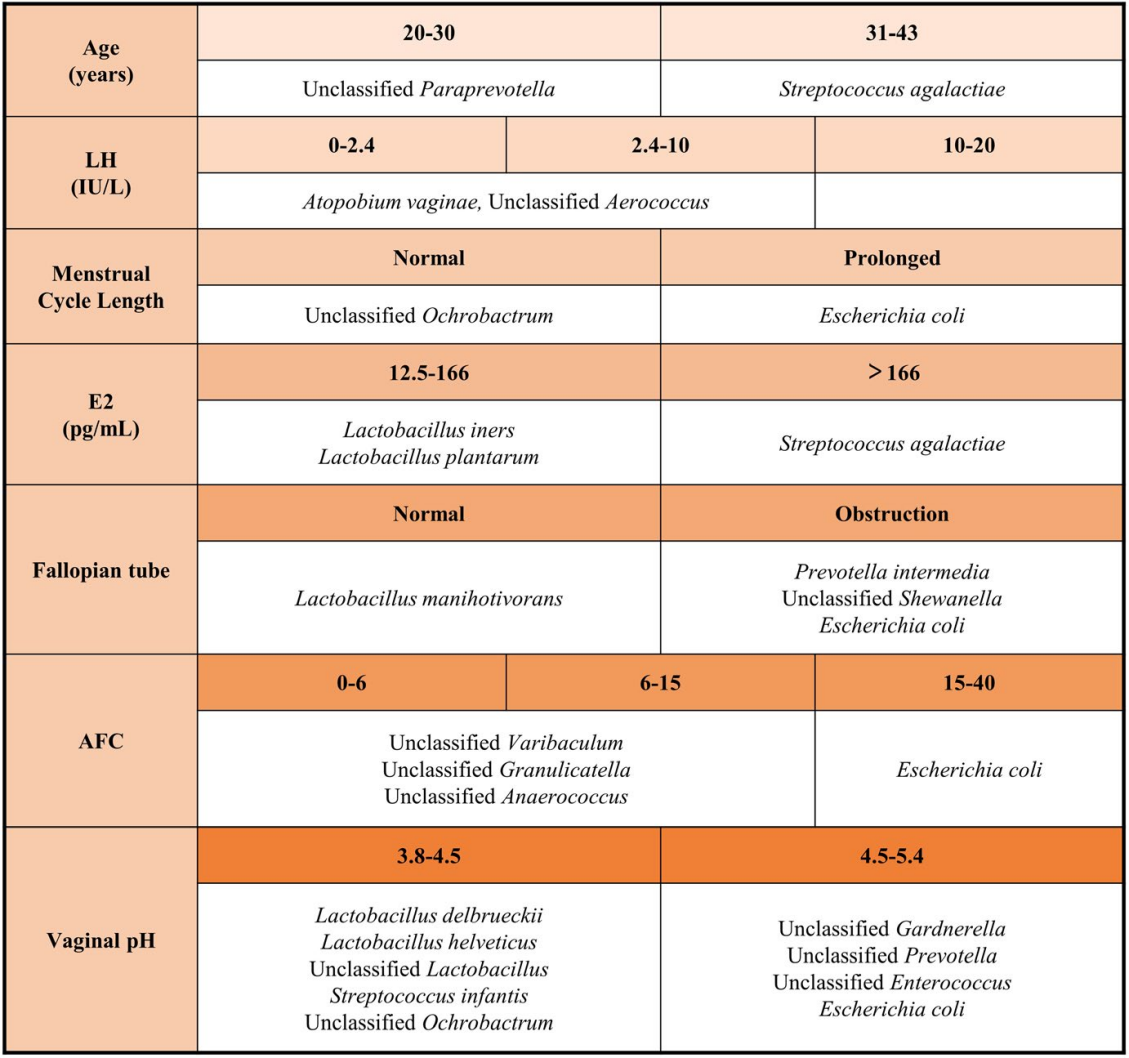
Summary table of specific vaginal species in each subgroup of fertility factors. A summary table based on the results above, listing the specific vaginal species between/among the different levels of the clinical indexes in women of reproductive age. E2, estradiol. LH, luteinizing hormone. AFC, antral follicle count

We have concluded the results above in Figure 5, showing the specific vaginal species between/among subgroups of each fertility factor:

Compared with the younger women, the older women had a higher relative abundance of *Streptococcus agalactiae* in the vagina and a lower relative abundance of unclassified *Paraprevotella*. In women with higher levels of E2, there were fewer *Lactobacillus iners* and *Lactobacillus plantarum* in the vagina but more *Streptococcus agalactiae*. For women with the basal LH level < 10 IU/L, the abundances of *Atopobium vaginae* and unclassified *Aerococcus* in the vagina were significantly higher than that in women with basal LH level > 10 IU/L.

Moreover, women with tubal obstruction had a higher relative abundance of *Escherichia coli*, *Prevotella intermedia*, and unclassified *Shewanella* in the vagina compared with the women with the normal fallopian tubes. Meanwhile, there were more *Lactobacillus manihotivorans* in the latter group compared to the previous group. It was worth noting that women with prolonged menstrual cycle also had more *Escherichia coli* and less unclassified *Ochrobactrum* compared to women with normal menstrual cycles.

We also found out that when the number of AFC was at different levels, the abundances of vaginal bacterial species in women of reproductive age also had specific differences. For women whose AFC was between 0‐6 and 6‐15, the relative abundances of unclassified *Varibaculum*, unclassified *Granulicatella*, and unclassified *Anaerococcus* in the vagina were higher than that in AFC > 15 group. Meanwhile, the relative abundance of *Escherichia coli* in the posterior group was higher than in the former two groups. Women with higher vaginal pH had less *Lactobacillus delbrueckii*, *Lactobacillus helveticus*, unclassified *Lactobacillus*, *Streptococcus infantis*, and unclassified *Ochrobactrum* in the vagina, while the colonization of unclassified *Prevotella*, unclassified *Enterococcus*, unclassified *Gardnerella,* and *Escherichia coli* increased compared to the women with normal vaginal pH.

## 4 DISCUSSION

The results above suggested that the fertility factors associated with reproductive endocrine and the reproductive tract environment could affect the vaginal microbiome in women of reproductive age. The relative abundances of several vaginal bacterial species were significantly different due to the host's age, levels of basic sexual hormones, antral follicle count, fallopian tube patency, menstrual cycle length, and vaginal pH.

We found that in older women, or women with higher levels of basal E2, the abundance of *Streptococcus agalactiae* in the vagina was higher than the normal group. *Streptococcus agalactiae*, also known as Group B *Streptococcus* (GBS), is an important pathogen in aerobic vaginitis. It rarely causes infections in healthy adults; however, occasionally it may cause morbidity in older women, pregnant women, or the patients with underlying predisposing conditions.^25^

Elderly adults account for >40% of persons with invasive GBS disease in the United States, colonized at vaginal and rectal sites. It causes skin and soft‐tissue infections, and bacteremia with no identified focus.^26^, ^27^ In pregnant and postpartum women, GBS is a frequent cause of asymptomatic bacteriuria, urinary tract infection, and upper genital tract infection et al.^28^, ^29^ It is noted that invasive maternal infection with GBS is associated with pregnancy loss and preterm delivery.^30^, ^31^

In the early follicular phase of women of reproductive age, high levels of basal E2 (>60‐80 pg/mL) with normal FSH levels may indicate a poor ovarian response. Early follicular E2 levels are better used with basal FSH to assess ovarian reserve.^32^ However, the relationship between GBS and the ovarian response has not been studied, and our study was the first to find that GBS infection might be associated with the decline of ovarian function.

In women with higher levels of basal E2 (>166 pg/mL), there were fewer *Lactobacillus iners* and *Lactobacillus plantarum* in the vagina than women with normal levels of basal E2 (12.5‐166 pg/mL). *Lactobacillus iners* is the most prevalent lactobacilli in most women.^33^ However, *L iners* lacks the gene coding for D‐lactic acid dehydrogenase,^34^ only producing the L‐lactic acid isomer.^35^ *Lactobacillus iners* dominated vaginal community type seems to be less stable or more in transition than the other community types and more associated with vaginal dysbiosis.^36^ Our results suggested that high level of basal E2 may have an impact on the colonization of *Liners*.

*Lactobacillus plantarum* strains produce anti‐infective agents, including hydrogen peroxide, and are able to co‐aggregate efficiently with vaginal pathogens.^8^ It helps treat vulvovaginal candidiasis and bacterial vaginosis, and restore a normal vaginal microbiota.^37^, ^38^

Moreover, it was first found that the abundances of *Atopobium vaginae* and unclassified *Aerococcus* in the vagina were correlated with the basal LH level. LH activity had previously been deemed potentially detrimental to reproductive function.^39^ However, Sun et al^40^ found that a high basal LH level did not have any adverse effects on oocyte quality and embryo quality and did not lead to poor outcomes of IVF/ICSI treatment. Moreover, more embryos and more top‐quality embryos developed in the group with LH > 10 mIU/mL than in the other groups.

The genera *Prevotella*, *Atopobium*, and *Gardnerella* were associated with bacterial vaginosis (BV), which may be linked to an increased risk of preterm labor.^11^ Abnormal vaginal microbiota, such as a high abundance of *Gardnerella vaginalis* or *Atopobium vaginae*, may negatively affect the clinical pregnancy rate in IVF patients.^41^

Based on our results, it was also first discovered that the number of antral follicles was also associated with the colonization of genera *Granulicatella*, *Anaerococcus*, and *Varibaculum* in the vagina. Previous researches have shown that the relative abundances of genera *Granulicatella* and *Anaerococcus* were increased in bacterial vaginosis patients.^42^ *Varibaculum cambriense* has been isolated from intrauterine devices and human vagina and abscess specimens.^43^

It was interesting to notice that women with AFC > 15 or prolonged menstrual cycle all had a higher abundance of *Escherichia coli* in the vagina. When the number of antral follicles is too high, it is often suggested that the subject has high levels of serum androgen, resulting in hyperandrogenic anovulation.^44^ Prolonged menstrual cycle, increased number of antral follicles, and the elevated serum androgen all suggest the occurrence of polycystic ovary syndrome (PCOS). Studies have found that the abundance of *Escherichia/Shigella* was higher in the intestinal flora of PCOS patients compared with the control group, and it was positively correlated with the levels of serum testosterone of the subjects,^45^ suggesting the pathological relationship between *Escherichia/Shigella* and PCOS. A study investigated the inflammatory associated microbial dysbiosis that occurred in a primate model of PCOS. They found that both diet and androgen treatment result in alterations in the genital tract microbiome, such as *Sneathia* and *Mobiluncus*, suggesting that inflammatory associated cervicovaginal microbiome dysbiosis plays a role in adverse fertility and pregnancy outcomes seen with PCOS.^46^

Moreover, we also found that women with tubal obstruction had a higher abundance of *Escherichia coli*, *Prevotella intermedia*, and unclassified *Shewanella* in the vagina than women with normal fallopian tubes. A previous study showed that the women in the stage 3/4 endometriosis group had more *Escherichia/Shigella* in the cervical and stool microbiome,^47^ which suggested that *Escherichia coli* may cause inflammatory reactions both in the reproductive tract and intestine. Virulence factor analysis showed that vaginal *Escherichia coli* was a reservoir along the “fecal‐vaginal‐urinary/neonatal” course of transmission in the extraintestinal *E coli* infections.^48^ Moreover, the vaginal colonization by *E coli* was regarded as a risk factor for very low birth weight delivery and other perinatal complications.^49^ *E coli* was also present in the vagina of subjects with elevated vaginal pH. The results above suggested that *E* *coli* might have a negative impact on female reproductive tract infection, tubal obstruction, and fertility, which deserves serious attention.

*Prevotella intermedia* was isolated from endometrial cultures of women with acute salpingitis.^50^ Since the pelvic inflammatory disease is caused by microorganisms that ascend from the vagina and endocervix to the endometrium and fallopian tubes, *P intermedia* may cause inflammation of the endometrium and fallopian tubes in succession, affecting female reproductive health.

Studies also have found that *Shewanella* colonized in the female reproductive tract in women of reproductive age, including the peritoneal fluid from the pouch of Douglas (5.6%), the endometrium (3.38%), and the fallopian tubes (1.81%).^51^ There are several reports describing the soft‐tissue and wound infection caused by *Shewanella* spp.^52^ However, the effects of *Shewanella* on the female reproductive tract have not been thoroughly studied.

The results above suggest that vaginal bacteria may affect the patency of fallopian tubes by causing the female genital infection.

In addition, we found that women with normal menstrual cycles have more unclassified *Ochrobactrum* in the vagina than women with the prolonged menstrual cycle, which is an emerging opportunistic pathogen in immunocompromised patients.^53^ It should be noted that although subjects with mycoplasma or chlamydia infection were excluded according to the clinical laboratory tests, some subjects were found to had *Mycoplasma* or *Ureaplasma* colonized in the vagina with a relative abundance of <5%. This may be due to the higher sensitivity and precision of 16S rRNA sequencing.

The vaginal microbiome played a central role in protecting and influencing the health of the vaginal environment. It was clinically meaningful to study how the fertility factors affect the vaginal microbiome in women of reproductive age. These results were progressive and mutually confirmed, proving that the pathological state of reproductive endocrine and genital tract could affect the composition of the vaginal microbiome. It also suggested that we can simultaneously detect the vaginal microbiome as an auxiliary assessment when testing the fertility‐related clinical indexes for the evaluation of female fertility. However, the gonadal axis involves a complex interaction between the hypothalamus, pituitary gland, and the gonads. These glands often act in concert to regulate development, reproduction, aging, and many other body processes. In the analysis of clinical trials, it was difficult to completely control the multiple variables and only study the effect of one single variable.

It should be noted that the levels of basic sexual hormones were measured during the early follicular phase (day 1‐5), not fixed on the 3rd of the menstrual cycle. It may have slight fluctuations during this period, which may affect the accuracy of correlation analysis between the reproductive endocrine and vaginal microbiome. Furthermore, the samples of vaginal microbiome were obtained during the menstrual interval. Though the previous study showed that the vaginal bacterial communities seemed resilient or almost invariant during the menstrual cycle,^54^ the sex hormone levels on the day of sampling may have an effect on the vaginal microbiome. Moreover, with the small sample size, cautions must be applied, and the actual relevance and clinical significance remain to be verified through a series of clinical control studies and physiological experiments.

## 5 CONCLUSIONS

The fertility factors associated with reproductive endocrine and the genital tract environment had effects on the vaginal microbiome in women of reproductive age. The bacterial species *Escherichia coli*, *Lactobacillus iners*, *Streptococcus agalactiae*, *Prevotella intermedia*, etc could be used as biomarkers to reflect the pathological state of reproductive endocrine and genital tract.

## CONFLICT OF INTEREST

The authors have no conflicts of interest to declare.

## HUMAN SUBJECTS APPROVAL

This study was approved by the Ethical Committee of Ren Ji Hospital, Shanghai Jiao Tong University School of Medicine (Approval No. 2016012904).

## PUBLIC SHARING OF DATA

Sequence data were archived in the NCBI Sequence Read Archive (SRA) under accession number SRP136384.
