# Supplementary figures and images for: Fertility factors affect the vaginal microbiome in women of reproductive age
Source: Am J Reprod Immunol. 2020 Jan 21;83(4):e13220. doi: 10.1111/aji.13220 (PMC7078941; doi:10.1111/aji.13220)

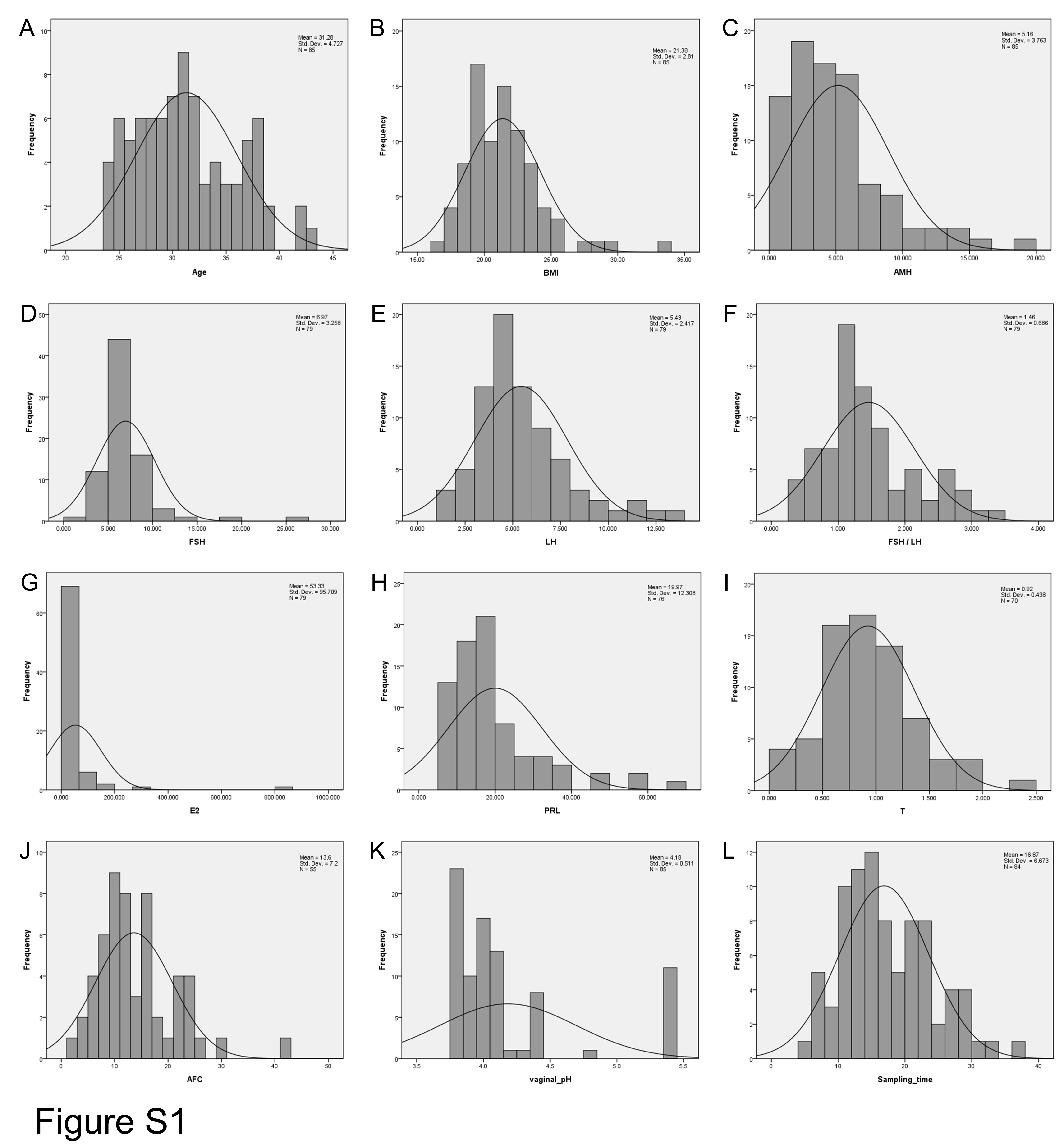

Supplement: Supplementary file 1 [file AJI-83-e13220-s001.tif]
